# Complete response of advanced HER2-amplified lung adenocarcinoma to cadonilimab combined with disitamab vedotin: a case report

**DOI:** 10.3389/fonc.2025.1587210

**Published:** 2025-11-03

**Authors:** Yue Li, Keru Ma, Hao Wang, Zongying Liu, Zhuying Li

**Affiliations:** ^1^ Department of Respiratory Medicine, First Affiliated Hospital of Heilongjiang University of Chinese Medicine, Harbin, Heilongjiang, China; ^2^ Department of Breast Surgery, Harbin Medical University Cancer Hospital, Harbin, China; ^3^ Department of Medical Oncology, Harbin Medical University Cancer Hospital, Harbin, China

**Keywords:** HER2 amplification, immunotherapy, complete response, non-small cell lung cancer, case report

## Abstract

**Background:**

Human epidermal growth factor receptor 2 (HER2) gene amplification in lung adenocarcinoma is associated with aggressive tumor behavior and poor prognosis. Currently, there is no standard targeted therapy for HER2-amplified lung cancer.

**Case summary:**

A 60-year-old male presented with a mass in the right cervical spine area. Imaging and pathological examinations confirmed stage IV (T4N3M1) lung adenocarcinoma with metastases to both lungs, multiple lymph nodes, the pleura, the left adrenal gland, and the meninges. Genetic testing revealed HER2 gene amplification and low programmed death-ligand 1 (PD-L1) expression (tumor proportion score <1%). The patient declined conventional chemotherapy due to concerns about side effects. With his informed consent, he was treated with a combination of cadonilimab (a PD-1/cytotoxic T-lymphocyte antigen 4 bispecific antibody) and disitamab vedotin (an anti-HER2 antibody–drug conjugate), administered intravenously every 3 weeks. After nine treatment cycles over 6 months, imaging assessments showed complete disappearance of the pulmonary lesions, and the achieved complete response (CR) persisted for at least 7 months. The treatment was well-tolerated, and the patient maintained an excellent performance status (Eastern Cooperative Oncology Group score of 0) without significant adverse effects.

**Conclusion:**

Treatment with cadonilimab and disitamab vedotin induced a sustained complete response in a patient with advanced HER2-amplified lung adenocarcinoma who declined chemotherapy. Thus, this combination therapy may offer a promising, effective, and well-tolerated treatment alternative for similar patients. Further clinical trials are warranted to validate these findings and potentially establish a new standard of care for this subset of lung cancer patients.

## Introduction

Lung cancer remains one of the leading causes of cancer-related morbidity and mortality worldwide, with non-small cell lung cancer (NSCLC) accounting for approximately 85% of all cases (1). Lung adenocarcinoma is the most prevalent of the NSCLC subtypes, and the prognosis for patients with advanced-stage lung adenocarcinoma is generally poor. Traditional chemotherapy and radiotherapy continue to be the standard treatments for lung adenocarcinoma, but some targeted therapies have shown promise, particularly for tumors harboring specific genetic alterations (2).

Alterations in the human epidermal growth factor receptor 2 (HER2) gene, including mutations and amplifications, have been detected in some cases of lung adenocarcinoma and shown to be associated with aggressive tumor behavior and poor prognosis (3). Despite this, no standardized treatment has been established to specifically target HER2 alterations in lung cancer, and the efficacy of anti-HER2 therapies currently approved for breast cancer in this context remains under investigation (4).

Immunotherapy has revolutionized the treatment landscape for NSCLC, with immune checkpoint inhibitors (ICIs) demonstrating significant survival benefits (5). Cadonilimab, a novel bispecific antibody targeting both programmed cell death protein 1 (PD-1) and cytotoxic T-lymphocyte antigen 4 (CTLA-4), has shown enhanced antitumor activity with a manageable safety profile in NSCLC cases in early-phase clinical trials (6). Disitamab vedotin, an antibody–drug conjugate targeting HER2, has exhibited efficacy in HER2-overexpressing tumors (7).

Here, we report a case of a 60-year-old male patient with advanced lung adenocarcinoma harboring HER2 gene amplification who refused conventional chemotherapy. He was treated with a combination of cadonilimab and disitamab vedotin and achieved a complete response (CR) that has persisted for more than 7 months. This case highlights the potential of combining immunotherapy with anti-HER2 therapy in the management of HER2-amplified NSCLC and suggests a promising therapeutic strategy that warrants further investigation.

## Case presentation

A 60-year-old male presented with a progressively enlarging mass on the right side of his neck that had been noticeable for more than 1 month. He was admitted to the hospital on March 29, 2024. The patient had no significant past medical history and maintained a good performance status, as indicated by a score of 0 on the Eastern Cooperative Oncology Group (ECOG) scale.

He first observed the swelling in his right neck region in February 2024, and initial imaging examinations were conducted in early March. At that time, ultrasound revealed a solid mass in the right cervical spine area. A biopsy of the mass confirmed poorly differentiated adenocarcinoma. Immunohistochemical staining showed positivity for cytokeratin (CK), CK7, and thyroid transcription factor-1 (TTF-1), along with weak positivity for Napsin A. The Ki-67 proliferation index was markedly high at 90%, indicating high proliferative activity of tumor cells. Ki-67 is a general marker of cell proliferation and is commonly used to assess the growth fraction of various tumors, but it is not specific to lung cancer.

Positron emission tomography-computed tomography (PET-CT) scanning was performed on March 5, 2024 and revealed an irregularly shaped mass near the hilum of the right upper lung lobe that measured approximately 3.5 × 2.6 cm. The PET-CT scans also showed multiple nodular lesions in both lungs, consistent with pulmonary metastases. Enlarged lymph nodes were observed in the bilateral supraclavicular regions and posterior diaphragmatic areas, with some nodes appearing indistinct from adjacent vascular structures. Additional findings on PET-CT scans included nodular lesions in both the pleura and left adrenal gland, suggestive of metastatic involvement of both of those tissues, as well as findings suspicious but not diagnostic of possible meningeal metastasis, without confirmatory cerebrospinal fluid (CSF) cytology or neurological symptoms.

Genetic testing on March 19, 2024 revealed amplification of the HER2 gene. The programmed death-ligand 1 (PD-L1) tumor proportion score (TPS) was less than 1%. The results of routine blood tests, including complete blood count and biochemical profiles, were within normal limits.

Based on the clinical, imaging, and pathological findings, the patient was diagnosed with stage IV (T4N3M1) right lung adenocarcinoma with metastases to both lungs, multiple lymph nodes (supraclavicular and mediastinal), the pleura, the left adrenal gland, and the meninges. Standard treatment options, including systemic chemotherapy, were discussed. However, the patient declined chemotherapy due to concerns about potential side effects and the impact on his quality of life. After thorough consideration and with informed consent from the patient, a novel off-label treatment regimen was initiated.

The combination of cadonilimab (a PD-1/CTLA-4 bispecific antibody) and disitamab vedotin (an anti-HER2 antibody-drug conjugate) was selected based on the rationale that targeting HER2 amplification with disitamab vedotin, alongside immune checkpoint inhibition with cadonilimab, would provide a dual approach to target tumor cells and enhance antitumor immunity. This combination was chosen to overcome the potential limitations of PD-L1 negativity and provide a more comprehensive treatment strategy. Both drugs were administered intravenously every 3 weeks.

On March 29, 2024, the patient began receiving the combination therapy. Over the subsequent 6 months, the patient completed nine cycles of the combination therapy. Regular monitoring included clinical evaluations, laboratory tests, and imaging studies to assess treatment efficacy and detect any adverse effects.

**Figure 1:**
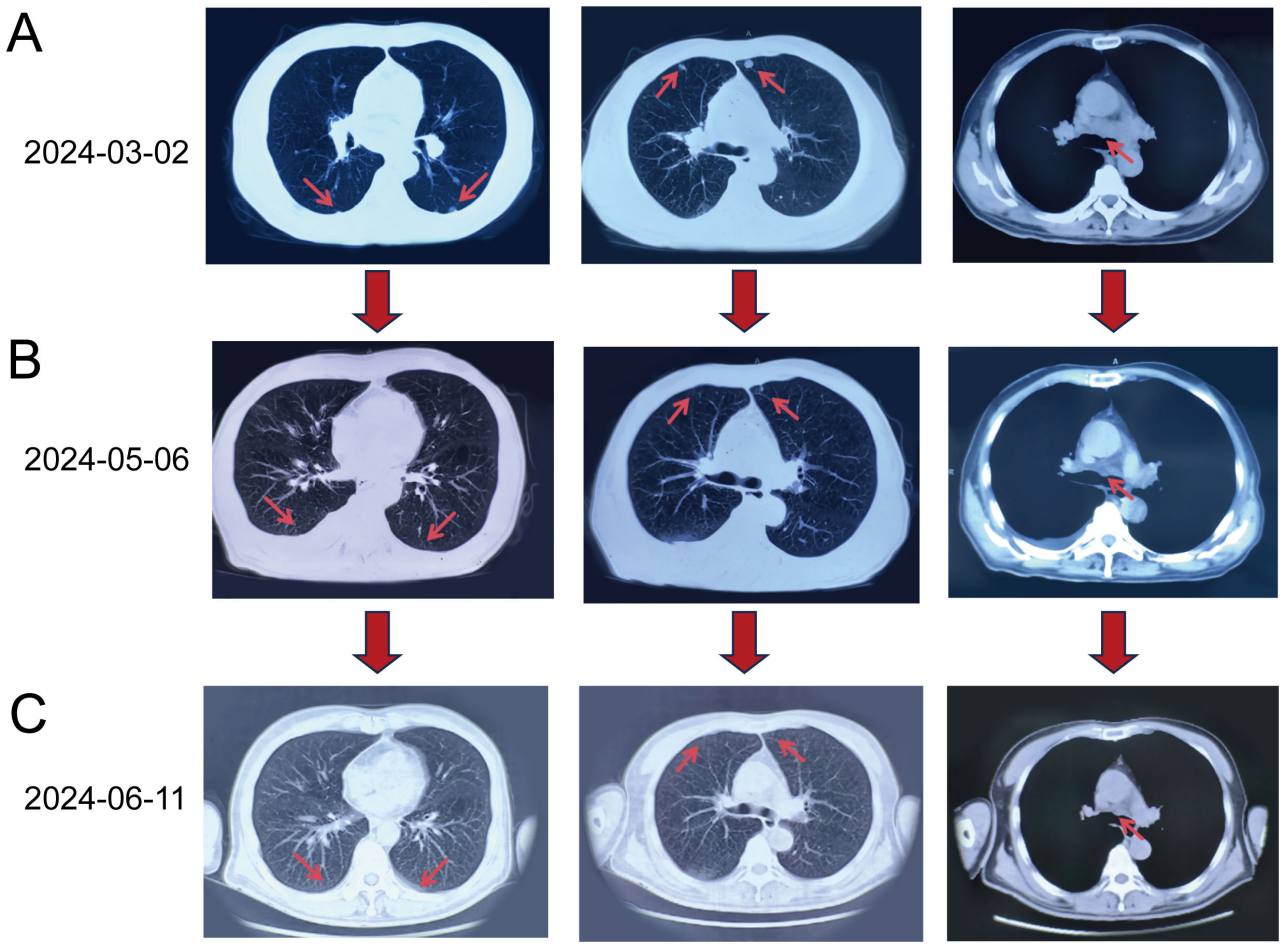
Chest CT scans obtained at different time points. **(A)** Baseline CT on March 2, 2024: Multiple nodular lesions (red arrows) in both lungs and enlarged mediastinal lymph nodes were visible. **(B)** Follow-up CT on May 6, 2024 (after four treatment cycles): Reduction in the size and number of pulmonary nodules (red arrows) and mediastinal lymph nodes (red arrow) was evident. **(C)** CT on June 11, 2024 (after six treatment cycles): Complete resolution of pulmonary nodules (red arrows) and further reduction in mediastinal lymph nodes (red arrow) were observed, indicating a complete response in the lungs.

To confirm the complete response (CR), we conducted multiple imaging evaluations, including chest CT and PET-CT scans, which covered all known metastatic lesions. Follow-up imaging at different intervals (May 2024 and June 2024) demonstrated the complete resolution of the right upper lobe mass and absence of pulmonary nodules. Chest CT scans performed at multiple intervals (**Figure 1**) supported these findings. Furthermore, the levels of tumor markers, including CEA and CA-125, were also monitored and showed significant reductions, indicating a favorable treatment response. These comprehensive evaluations support the conclusion of a CR, consistent with the RECIST guidelines.

**Figure 2:**
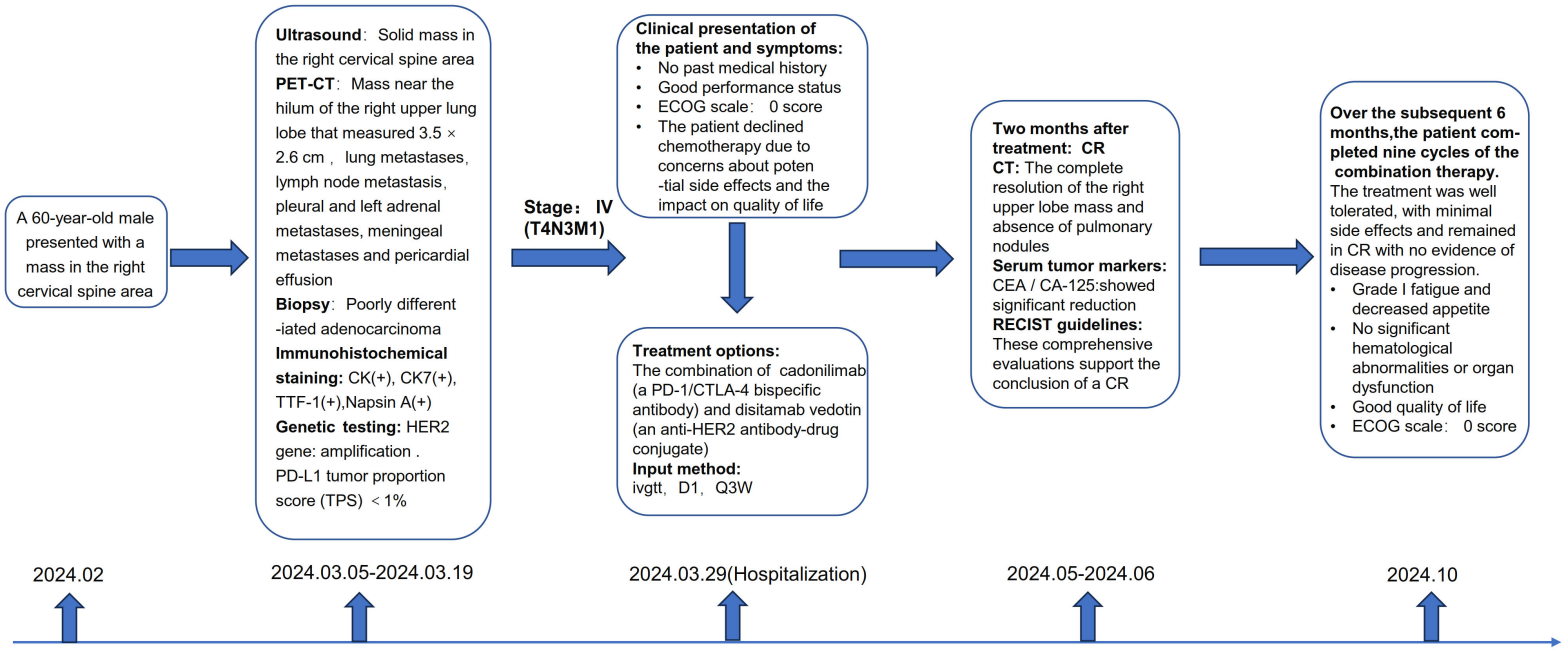
Schematic clinical presentation of the patient and symptoms.

Overall, the treatment was well-tolerated, with minimal side effects. The patient experienced grade I fatigue and decreased appetite, which were effectively managed with rest, supportive care, dietary modifications, and appetite stimulants. No significant hematological abnormalities or organ dysfunction were observed on laboratory tests throughout the treatment course. The patient reported a good quality of life during treatment, as he continued to engage in normal daily activities without significant limitations. Throughout the treatment period, he maintained an excellent performance status (ECOG score of 0), reflecting full activity without symptoms. At the time of the last follow-up in October 2024, he remained in complete remission with no evidence of disease progression. Regular follow-up visits and imaging studies are planned to monitor for any signs of recurrence (**Figure 2**).

## Discussion

This case demonstrates achievement of a significant and sustained complete response in a patient with advanced HER2-amplified lung adenocarcinoma who was treated with the combination of cadonilimab and disitamab vedotin, after declining conventional chemotherapy. The patient’s extensive disease at diagnosis, including pulmonary, lymphatic, pleural, adrenal, and possible meningeal metastases, coupled with low PD-L1 expression and a high Ki-67 index, portended a poor prognosis (8). Despite these factors, the patient achieved and maintained complete remission for more than 7 months with minimal toxicity, suggesting that this novel combination therapy may offer a potential therapeutic avenue for similar cases.

HER2 (ERBB2) gene alterations, such as mutations and amplifications, are identified in approximately 2–4% of NSCLC cases, predominantly among adenocarcinoma cases. HER2 amplification is associated with aggressive tumor behavior and poorer clinical outcomes. Unlike breast cancer, for which HER2-targeted therapies are well-established, there is no standard treatment for HER2-amplified NSCLC, and patients often have limited options beyond conventional chemotherapy (9).

Disitamab vedotin is a novel antibody-drug conjugate (ADC) that targets HER2. It consists of a humanized anti-HER2 antibody linked to the cytotoxic agent monomethyl auristatin E (MMAE) and has shown promising efficacy in HER2-expressing gastric and urothelial cancers (10–13). The ADC binds to HER2-overexpressing tumor cells, and upon its internalization, it releases MMAE to induce cell cycle arrest and apoptosis. Preliminary studies in lung cancer patients have suggested that disitamab vedotin may be effective in cases with HER2 alterations, but the relevant clinical data remain limited (10).

Immunotherapy with immune checkpoint inhibitors (ICIs) targeting PD-1/PD-L1 has revolutionized the treatment of advanced NSCLC. However, cases with low or negative PD-L1 expression often derive less benefit from monotherapy with ICIs (11).

In this case, the patient’s low PD-L1 expression could have been a significant challenge for effective immunotherapy. However, treatment with the combination of cadonilimab and disitamab vedotin might overcome this limitation. Cadonilimab is a bispecific antibody that targets both PD-1 and CTLA-4, effectively blocking two critical immune checkpoints that tumors exploit to evade immune surveillance. By inhibiting both PD-1 and CTLA-4, cadonilimab enhances T-cell activation and reprograms the immune microenvironment, even in the absence of high PD-L1 expression. While PD-L1 expression is typically used as a biomarker to predict the efficacy of PD-1 inhibitors, cadonilimab’s dual checkpoint inhibition may bypass the need for elevated PD-L1 levels, potentially restoring antitumor immunity (14). Additionally, HER2 amplification in lung adenocarcinoma is linked to aggressive tumor behavior and poor prognosis. HER2 overexpression can promote immune evasion by suppressing tumor-infiltrating lymphocytes (TILs) and activating immune checkpoint pathways. Disitamab vedotin, an anti-HER2 antibody-drug conjugate (ADC), targets HER2-positive tumor cells directly. It also has the potential to enhance tumor antigen presentation and foster a more immune-permissive microenvironment. The release of tumor-associated antigens and pro-inflammatory signals from the ADC may help convert a “cold” tumor microenvironment into a “hot” one, thus improving the efficacy of immunotherapy (15).

The combination of ADCs with immunotherapy represents an emerging strategy designed to generate synergistic antitumor effects. ADC-induced tumor cell death releases neoantigens and pro-inflammatory signals, promoting dendritic cell activation and T-cell priming. This process may enhance the responsiveness of the tumor microenvironment to immunotherapy, as demonstrated by disitamab vedotin’s role in antigen presentation and cadonilimab’s amplification of the immune response (16).

The patient’s refusal of chemotherapy in this case underscores the necessity for alternative treatments that are both effective and tolerable. The combination therapy provided substantial clinical benefits without causing any of the significant side effects typically associated with chemotherapy (17). The patient’s excellent performance status and quality of life throughout the treatment period highlight the potential of this regimen as a viable option for patients who are ineligible for or decline chemotherapy.

To date, there have been few studies on the use of disitamab vedotin to treat NSCLC, and data are lacking regarding the efficacy of combining a HER2-targeted ADC with immunotherapy in this setting. Previous studies involving the use of other anti-HER2 agents, such as trastuzumab and ado-trastuzumab emtansine (T-DM1), have shown modest benefits in NSCLC. Moreover, the addition of ICIs to targeted therapies has been explored in the treatment of other cancers. Although the results have suggested enhanced efficacy, robust clinical evidence in NSCLC is still needed. This present case contributes valuable clinical insight into the potential effectiveness of this combination therapy as treatment for HER2-amplified NSCLC (18–20).

While the outcome in the presented case is encouraging, this is only a single case report. Larger clinical studies are necessary to evaluate the safety, efficacy, and generalizability of combining cadonilimab with disitamab vedotin in the treatment of NSCLC with HER2 amplification. Additionally, the identification of biomarkers that can aid the prediction of treatment response will be crucial for selection of the patients who are most likely to benefit from this therapy. The optimal duration of treatment and management of potential long-term adverse effects also require further investigation. As the patient remains in remission, considerations regarding maintenance therapy versus discontinuation need to be addressed in future studies.

## Conclusion

In the presented case, treatment with the combination of cadonilimab and disitamab vedotin induced a sustained complete response in a patient with advanced HER2-amplified lung adenocarcinoma who declined chemotherapy. The treatment was well-tolerated, and the patient’s quality of life remained good throughout the treatment period. These findings suggest that this combination therapy may offer a promising alternative for patients with similar clinical profiles. Further clinical trials are warranted to validate the results achieved in this case, and the results may serve to inform a new standard of care for this subset of NSCLC patients.

## Data Availability

The original contributions presented in the study are included in the article/supplementary material. Further inquiries can be directed to the corresponding author.
